# Taxonogenomic analysis of the Xanthomonas translucens complex leads to the descriptions of Xanthomonas cerealis sp. nov. and Xanthomonas graminis sp. nov.

**DOI:** 10.1099/ijsem.0.006523

**Published:** 2024-09-19

**Authors:** James T. Tambong, Renlin Xu, Maria Constanza Fleitas, Randy Kutcher

**Affiliations:** 1 Ottawa Research and Development Centre, Agriculture and Agri-Food Canada, Ottawa, ON, Canada; 2 Department of Plant Science, University of Manitoba, Winnipeg, MB, Canada; 3 Department of Plant Sciences & Crop Development Centre, University of Saskatchewan, Saskatoon, SK, S7N 5A8, Canada

**Keywords:** average nucleotide identity, barley, DNA-DNA hybridization, forage, pathovars, taxonomy, wheat, whole-genome sequence, *Xanthomonas translucens*

## Abstract

The pathovar-based taxonomy of the *Xanthomonas translucens* group is very confusing due to an overlap of plant host ranges and level of host specificity. Here, whole-genome sequence-based parameters (digital DNA–DNA hybridization and blast-based average nucleotide identity), phylogenomic, biochemical and phenotypical data were used to taxonomically analyse the 11 known pathovars of the *X. translucens* complex. This polyphasic approach taxonomically assigned the 11 pathovars of * X. translucens* complex into three distinct species, two of which are new: *X. translucens*, *X. cerealis* sp. nov. and *X. graminis* sp. nov. *X. translucens* consists of three pathovars: pv. *translucens* (=pv. *hordei*), pv. *pistaciae* strain A ICMP 16316^PT^ and pv. *undulosa* (=pv. *secalis*). *X. cerealis* sp. nov. encompasses the pv. *cerealis* strain LMG 679^PT^ and pv. *pistaciae* strain B ICMP 16317^PT^ with genome similarity of 92.7% (dDDH) and 99.0% (ANIb) suggesting taxonomically similar genotypes. The other new species, *X. graminis* sp. nov., consists of the remaining five designated pathovars (pv. *graminis*, pv. *arrhenatheri,* pv. *poae*, pv. *phleipratensis* and pv. *phlei*) with highly variable dDDH and ANIb values ranging from 74.5 to 93.0% and from 96.7 to 99.2%, respectively, an indication of a very divergent taxonomic group. Only strains of pvs. *phlei* and *phleipratensis* showed the highest genomic similarities of 93.0% (dDDH) and 99.2% (ANIb), suggesting synonymic pathovars as both infect the same plant hosts. The dDDH and ANI data were corroborated by phylogenomics clustering. The fatty acid contents were similar but the type strain of *X. graminis* sp. nov. exhibited 20% less C_15 : 0_ iso and 40% more C_17 : 0_ iso fatty acids than the other species. Based on phenotypic, biochemical and whole-genome sequence data, we propose two new species, *Xanthomonas cerealis* sp. nov. and *Xanthomonas graminis* sp. nov. with type strains LMG 679^T^ (=NCPPB 1944^T^) and LMG 726^T^ (=NCPPB 2700^T^), respectively.

## Introduction

Species of the genus *Xanthomonas* Dowson 1939 (*Xanthomonadaceae*, Gammaproteobacteria) are a versatile group of bacteria isolated from a variety of plant hosts. *Xanthomonas* species are reported to cause diseases on economically relevant dicot and monocot species [1,2] ranging from cultivated crops such as tomato, barley, rice, to trees, for example, pistachio. The genus *Xanthomonas* consists of 34 validly published species (https://lpsn.dsmz.de/genus/xanthomonas accessed 30 May 2024 [3]). Some species of *Xanthomonas* show a high degree of host plant specificity [2], leading to a plenitude of pathovar designations. This issue was compounded by the ‘new’ host / ‘new’ species rule implemented before the 1940s [4] leading to a large number of poorly identified phytopathogenic species assigned to the pathovar level, especially in the genera*Pseudomonas* and *Xanthomonas*.

One of these species with pathovar-level assignments is *Xanthomonas translucens*. Strains of the *X. translucens* complex cause bacterial leaf streak (BLS) and black chaff diseases on cereal crops and wilt in forage and turfgrass [5,8]. Since these pathogens can be seed-borne [9], they are an A2 phytosanitary concern for international trade and exchange of germplasm (EPPO Data Sheets on Quarantine Pests). The current nomenclature and taxonomy of the *X. translucens* complex is based on the pathovar concept that is defined by distinctive pathogenicity to one or more plant hosts [10]. The *X. translucens* complex consists of 11 pathovars, viz *translucens*, *undulosa*, *cerealis*, *hordei*, *secalis*, *graminis*, *phlei*, *phleipratensis, arrhenatheri*, *poae*, and *pistaciae*. Pathovars *translucens*, and *hordei* are reported to be synonyms and same for pvs *undulosa* and *secalis*. Strains of pathovars *translucens*, *hordei*, *undulosa*, *secalis*, and *cerealis* cause BLS and glume black chaff disease on small grains and some grasses. With the exception of pv. *pistaciae* which causes disease on pistachio plants, the other pathovars are the causal pathogens of the bacterial wilt on forage grasses.

*Xanthomonas translucens* pv. *pistaciae* Giblot-Ducray *et al*. (2009) is the causal agent of the bacterial dieback disease (BDD) of pistachio (*Pistacia vera*, family *Anacardiaceae*) in Australia. BDD was first reported by Taylor and Edwards [11], and it is characterized by decline, trunk lesions, xylem staining and excessive resin exudation as well as inconsistent foliar symptoms [12,13]. The pathovar is reported to have two distinct genotypic pathotypes, strain A (ICMP 16316) and strain B (ICMP 16317), based on ITS sequence analysis, rep-PCR, pathogenicity and host-range studies [14].

Increasingly, the pathovar concept has become unreliable and confusing due to the level of host plant specificity and the overlap of plant ranges among the pathovars [7,8, 15]. For example, pv. *undulosa* is pathogenic on wheat, barley, rye grasses and triticale (x Triticosecale) while pv. *translucens* is specific on barley with almost identical field disease symptoms; and pv. *cerealis* is pathogenic to barley, oat, wheat, and bromegrass [4,8, 16,19]. There is a need for an improved taxonomic nomenclature of the *X. translucens* pathovars. Several classical bacteriological techniques such as protein assays, biochemical, physiological and serological tests as well as DNA-based fingerprinting techniques, for example, restriction fragment length polymorphism did not provide taxonomic clarity of the pathovars [20,25].

Taxogenomic analysis of whole-genome sequences (complete and draft chromosomes) data is increasingly employed to better elucidate the DNA relatedness and evolutionary relationships of bacterial species [26,28]. Genome-based studies of a few *Xanthomonas* species have provided more clarity to taxonomic relatedness, geographic groupings and even distinction of pathovars [29,31]. Harrison *et al*. [32] used phylogenomic analysis to transfer 20 pathovars from *Xanthomonas campestris* into *Xanthomonas euvesicatoria*. Genome-based data led Constantin *et al*. [33] to reclassify *Xanthomonas perforans* and *Xanthomonas alfalfae* as pathovars of *X. euvesicatoria*. Using whole-genome sequence data, Zarei *et al*. [34] refined the taxonomy of strains of * X. arboricola* pv. *guizotiae* and *X. arboricola* pv. *populi* proposing the elevation of the two pathovars to species, ‘*X. guizotiae*’ sp. nov. and ‘*X. populina*’ sp. nov, respectively. No comprehensive taxonomic studies have been conducted on the *X. translucens* complex to provide some clarity. Analyses based on multilocus sequence analysis (MLSA) of housekeeping genes or limited genome-based parameters have been published [7,35]. Langlois *et al*. [7] used MUMmer-based average nucleotide identity (ANIm) values and MLSA of 12 housekeeping genes (20 kb) to assign seven of the *X. translucens* pathovars into three main clusters: pvs. *undulosa* and *translucens*, pv. *cerealis*, pvs. *arrhenatheri*, *poae*, *graminis*, and *phlei*. Goettelmann *et al*. [36] analysed all the pathovars leading to the conclusion that these strains represent three genetically distinct clades based on ANI values. Tambong *et al*. [37] analysed whole genome sequence data of the 11 pathovars and concluded that the three genetically distinct clades are valid and unique taxonomic groups. The primary objective of this study was to use whole genome-based digital DNA–DNA hybridization (dDDH), blast-based average nucleotide identity (ANIb), phylogenomics, physiological and biochemical data to propose elevating * X. translucens* pv. *cerealis* and *X. translucens* pv. *gramini*s to *Xanthomonas cerealis* sp. nov. and *X. graminis* sp. nov., respectively. The 11 pathovars were delineated into three distinct species: (1) *X. translucens* consisting of pvs. *translucens* (=pv. *hordei*), and *undulosa* (=pv. *secalis*), *pistaciae* strain A ICMP 16316; (2) *X. graminis* sp. nov. (pvs *graminis*, *arrhenatheri*, *poae*, *phlei* (=*phleipratensis*); and (3) *Xanthomonas cerealis* sp. nov. (pvs. *cerealis* and *pistaciae* strain B ICMP 16317). The secondary objective was to validate the affiliations of strains A and B of pv. *pistaciae* to two different species by comparative genomics (orthologous gene analysis) and pathogenicity on barley and wheat. Strain pathogenicity on barley and wheat is routinely used to differentiate strains of pv. translucens from pvs. *undulosa* and *cerealis*. Pathogenicity and orthologous gene analyses of strains A and B of pv. *pistaciae* validated the proposed taxonomic groupings to their respective proposed species, *X. translucens* and *X. cerealis* sp. nov. Pv. *pistaciae* strain A exhibited similar pathogenicity patterns as reported for *X. t. translucens*, pathogenic to barley but not wheat, suggesting that these strains belong to the same pathovar. Also, mild but observable symptoms were induced by pv. *pistaciae* strain B on both barley and wheat which is consistent with the pathology of pv. *cerealis*. In addition, the orthologous gene analysis showed the respective associated strain pairs sharing the highest number of protein families.

## Methods

### Bacterial strains and genome downloads

Two pv. *pistaciae* strains A (ICMP 16316) and B (ICMP16317) [38] were obtained from the National Collection of Plant Pathogenic Bacteria (NCPPB). Strains were revived as recommended and preserved at −80 °C in Luria–Bertani (LB; tryptone, 10 g l^−1^, yeast extract 5 g l^−1^, NaCl 10 g l^−1^) medium amended with 30% glycerol (v/v).

Whole- and draft-genome sequence data of the 12 pathotype strains of the *Xanthomonas translucens* complex and type strains of all validly published species of the genus *Xanthomonas* were downloaded from the GenBank database at www.ncbi.nlm.nih.gov/genome/ as previously reported [39].

### 16S rRNA; whole-genome comparison and phylogenomics

16S rRNA sequences of the 11 pathotypes were extracted from the whole-genome sequences using RNAmmer [40] and phylogenetic analysis performed as previously described [37].

ANIb [41] and the Genome-to-Genome Distance Calculator (dDDH [42]) were implemented to compare the whole genome data of the pathotypes. The species-level cut-off thresholds of 96 [41] and 70% [42] were used for ANIb and dDDH, respectively.

Phylogenomic analyses of the 12 strains of the pathovars of *X. translucens* and 36 other *Xanthomonas* species were done using Type (Strain) Genome Server (TyGS) [43,44]. TyGS implements the mash algorithm [45] using default parameters as reported previously [37]. Since whole genome sequences could contain artefacts, we used the 81 up-to-date bacterial core gene set pipeline (ubcg2 [46]) to validate the clustering patterns of the 11 pathovars and six closely related species identified on the TyGS-derived evolutionary tree. The ubcg-based tree was generated using RAxML-NG [47] with 1000 non-parametric bootstrap replicates. For orthologous gene analysis of the two pv. *pistaciae* strains A and B, the annotated protein output from prodigal [48] was used to identify and compare orthologs using the OrthoVenn3 platform [49].

### Pathogenicity of Xtp-A and Xtp-B on barley and wheat

The two pv. *pistaciae* strains were evaluated if they could induce pathogenic patterns. Growth chamber pathogenicity tests of * X. t*. pv. *pistaciae* A (Xtp-A) and *X. t*. pv. *pistaciae* strain B (Xtp-B) were performed as previously reported by Tambong *et al*. [37]. Briefly, spring wheat (cv. Hoffman), and Hulless barley (cv. CH2909.162.95) were grown in potting soil (75%:24%:1% Promix BX–black earth–lime) in a growth chamber at 25 °C. Plants were watered and fertilized regularly as required; and 21 days after sowing, five replicate plants were inoculated by the leaf infiltration method using a bacterial inoculum (1×10^8^ c.f.u. ml^−1^ in PBS) with PBS as negative control. Plants were observed daily for typical bacterial leaf streak symptoms.

### Fatty acid and phenotypic analyses

Whole cell fatty acids of the representative strains (LMG 726 (Xtg), LMG 679 (Xtc), LMG 876 (Xtt)) of the three proposed species were analysed as previously reported [50]. The extraction and analysis of fatty acid methyl esters were performed by Keystone Inc. (Alberta, Canada) according to the recommended protocol of midi Inc (The Sherlock Microbial Identification System (version 6.2) [51]. The Agilent 7890B gas chromatograph was used to generate the profiles and automatically identified by the midi TSBA 6 database.

Other standard bacteriological and biochemical characterizations were performed on the representative strains of the three proposed species as previously described [52]. Gram-reaction of the strains was determined using the 3% KOH assay [53]. The Biolog GEN III MicroPlate was used, in duplicates, to analyse 71 carbon sources and 23 chemical sensitivity assays, different pH levels, and salt tolerance as recommended by the manufacturer and data recorded 24 and 48 h after inoculation. Also, the API ZYM system (bioMérieux), in duplicates, was used to study the enzymatic activities of the strains as recommended by the manufacturer. Oxidase activity was tested using strips (Millipore-Sigma) [50,52].

## Results and discussion

### 16S rRNA phylogeny, genome-based DNA–DNA similarity and phylogenomics of pathovars of *Xanthomonas translucens* complex

The 16S rRNA maximum-likelihood tree grouped the *Xanthomonas* strains into two main groups (Fig. S1, available in the online Supplementary Material). All 12 strains from the pathovars clustered in group II with *X. hyacinthi*, *X. thieocola*, *X. bonasiae*, * X. albilineans* and *X. sacchari* as the closest relatives (Fig. S1). The 12 pathovar strains were divided into two subgroups (II-a and II-b; Fig. S1), but as expected could not delineate them into species due to the poor resolution power of 16S rRNA sequence data [27,50].

In recent years, using whole genome data, several strains and species of the genus *Xanthomonas* have been taxonomically revised. For example, Constantin *et al*. [33] genetically characterized the *X. axonopodis* complex; and Zarei *et al*. [34] refined the taxonomy of *Xanthomonas arboricola* leading to the descriptions of ‘*X. populina*’ sp. nov. and ‘*X. guizotiae*’ sp. nov., which were originally described as *X. arboricola* pv. *populi* by Vauterin *et al*. [54] and *X. campestris* pv. *guizotiae* (Yirgou 1964) Dye 1978, respectively.

To provide more clarity to the pathovar assignment of the *X. translucens*, we used genome-based data (dDDH, ANIb and phylogenomics analysis). Table 1 summarizes the results of the dDDH and ANIb values between the 12 known pathovar strains of *X. translucens* at the species level with cut-off values of 70 and 96%, respectively. The data presented here suggest three distinct species (Table 1). dDDH and ANIb values of pv. *translucens* were above the species level cut-off of 70 and 96% with only pv. *hordei* (90.1 and 98.78%), pv. *pistaciae* strain A (81.0 and 98.1%), pv. *secalis* (77.7 and 97.3%), and pv. *undulosa* (78.5 and 97.5%), respectively (Table 1; grey). These data suggest that these five pathovars taxonomically belong to the same species and designated as ‘true’ *X. translucens* in this study. Also, pv. *translucens*, exhibited mean dDDH and ANIb values above 83.0 and 97.0% with pvs. *hordei* (90.1 and 98.8%) and *pistaciae* strain A (83.8 and 98.0%), respectively, suggesting their genetic make-up is similar but not identical. Bradbury [55] and Bragard *et al*. [16] reported that pvs *translucens* and *hordei* are true synonyms. Pvs. *undulosa* and *secalis* had dDDH and ANIb values of 98.7 and 99.80%, confirming that these two pathovars are genetically similar and synonyms as previously reported [37] based on proteomes similarity of 95.8%. The whole genome sequences of the other pathovars and the five closest relative *Xanthomonas* species showed ANIb and dDDH values lower than the species-level cut-off values of 70 and 96%, respectively (Table 1). This suggests that the remaining pathovars cannot be valid members of the species *X. translucens* based on whole genome sequence data. For example, the dDDH and ANIb values between pv. *translucens* and pv. *cerealis* were only 59.3 and 94.5%, respectively (Table 1). Interestingly, the dDDH and ANIb values of 92.7 and 99.01%, respectively, were computed between pv. *cerealis* and pv. *pistaciae* strain B, values that are significantly above the 70 and 96% species-level delineation thresholds (Table 1). This indicates that pvs. *cerealis* and *pistaciae* strain B belong to the same but new species designated here as *Xanthomonas cerealis* sp. nov. Whole-genome sequence-based DNA–DNA values of the remaining pathovars (pv. *graminis*, pv. *arrhenatheri*, pv. *phlei*, pv. *poae*, and pv. *phleipratensis*) averaged 77.96% (dDDH) with a range of 74.5–93% within this subpopulation. The mean ANIb value of 97.33% with a range from 96.92 to 98.84% (Table 1) were all above the species-level cut-off values. This suggests that these five pathovars belong to the same species but taxonomically different from the ‘true’ *X. translucens* or the newly assigned *X. cerealis* sp. nov. We propose a new species, *Xanthomonas graminis* sp. nov., to encompass these five pathovars. Egli *et al*. [5] published a new species, *Xanthomonas graminis*, the causal agent of a new bacterial wilt on forage grass disease in Switzerland. However, the species name was not validly published in accordance with The Prokaryotic Code. Using wet-laboratory DNA–DNA hybridization (wDDH) data from previous studies, Vauterin *et al*. [54] proposed a new classification of 62 pathovars of *Xanthomonas campestris*. Vauterin *et al*. [51] reclassified *Xanthomonas campestris* pv. *graminis* to *Xanthomonas translucens* pv. *graminis*. One of the pathovars reclassified by Vauterin *et al*. [51] from *X. campestris* pv. *populi* to *X. arboricola* pv. *populi* has now been elevated to species ‘*Xanthomonas populina*’ based on whole genome sequence data [31]. Zarei *et al*. [31] also elevated *X. campestris* pv. *guizotiae* to ‘*X. guizotiae*’, which is one of the 62 pathovars that Vauterin *et al*. [51] reported to be unable to yet place in any species of *Xanthomonas* based on wDDH. This underscores the species level resolution power of whole genome sequence data in modern bacterial taxonomy and systematics.

**Table 1.** Pairwise digital DNA–DNA hybridization (dDDH; lower) and blast-based average nucleotide identity (ANIb; upper) among the 11 Xanthomonas transclucens pathovars as well as five closest Xanthomonas species

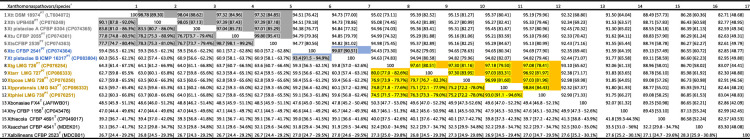 ­

∗dDDH and ANIb were computed as described by Meier-Kolthoff et al. (2013) [42] and Richter et al. (2016),[41] respectively.

†Xtt, *Xanthomonas translucens* pv. *translucens*; Xth, *X. t.* pv. *hordei*; Xt pistaciae, *X. t*. pv. *pistaciae*; Xtu, *X. t.* pv. *undulosa*; Xts, *X. t.* pv. *secalis*; Xtc, *X. t.* pv. *cerealis*; Xtg, *X. t.* pv. *graminis*; Xtarr, *X. t.* pv. *arrhenatheri*; Xtpoae, *X. t.* pv. *poae*; Xtppratensis, *X. t.* pv. *phleipratensis*; Xtphlei, *X. t.* pv. *phlei*; Xbonasiae, *Xanthomonas bonasiae*; Xhy, *Xanthomonas hyacinthi*; Xthiecola, *Xanthomonas thiecola*; Xsacchari, *Xanthomonas sacchari*; and Xalbilineans, *Xanthomonas albilineans*. Text color grey, members of the 'true' *X. translucen*s; blue, members of *X. cerealis sp.* nov.; and orange members of *X. graminis* sp. nov.

Giblot-Ducray *et al*. [38] assigned pv. *pistaciae* strains A and B as *X. transclucens* based on their wDDH values of 81±7 and 86±5% relative to *X. t*. pv. *translucens* LMG 876^PT^. Using genome-based dDDH, now considered as the new gold standard [56], pv. *pistaciae* strain A exhibited a mean value of 83.8% (species cut-off threshold of 70%) relative to *X. t*. pv. *translucens* LMG 876^PT^, thus, confirming its affiliation to *X. translucens*. Pv. *pistaciae* strain B, however, had a dDDH value of only 60.3% with *X. t*. pv. *translucens* LMG 876^PT^ which is below the 70% species cut-off threshold, but showed 93.4% with *X. t*. pv. *cerealis* CFBP 2541^PT^. A wDDH value of 84±5% was obtained between pv. *pistaciae* strains A and B [38] while dDDH showed 60.9%. It is unclear why Giblot-Ducray *et al*. [38] did not incorporate *X. t*. pv. *cerealis* in their wDDH experiments even though their *gyrB*-based phylogenetic tree showed it forming a distinct taxonomic clade (bootstrap value of 100%) with pv. *pistaciae* strain B ICMP 16317. Also, Giblot-Ducray *et al*. [35] recorded a wDDH value of 84±10% between *X. t*. pv. *poae* and pv. *pistaciae* strain A ICMP 16316 compared to a dDDH value of 63.5% with a confidence interval of 60.6–66.3% in our study. High standard deviations of wDDH data highlight its inherent drawbacks such as poor reproducibility between laboratories and high error [57,58].

**Fig. 1.**
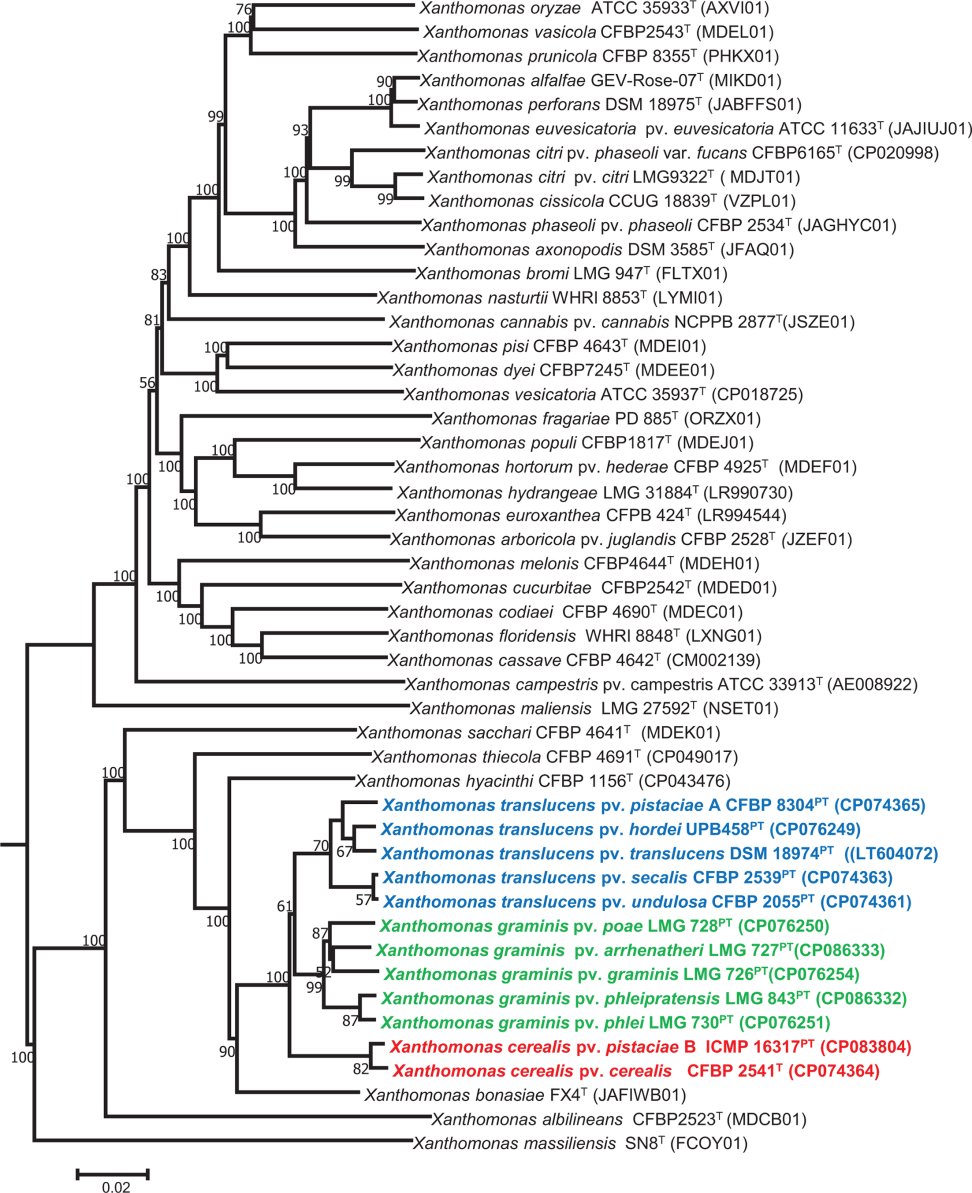
Evolutionary trees based on whole-genome distances [44] confirmed the delineation of three distinct species (blue, *X. translucens*; green, *X. graminis* sp. nov.; and red, *X. cerealis* sp. nov.) within the 11 pathovars of the *Xanthomonas translucens* complex. Bootstrap values >50% are shown at the nodes.

The dDDH and ANI results were strongly corroborated by phylogenomic analysis inferred on the TyGS platform [43,44] using GBDP-derived distances which provides genome-based taxonomy including prediction of novel taxa (Fig. 1). TyGS-based phylogenomic analysis of pvs. *undulosa, (=secalis*), *translucens (=hordei*), and *pistaciae* strain A confirmed that these strains belong to the same species, *X. translucens,* while members of *X. cerealis* sp. nov. (pvs. *cerealis* and *pistaciae* strain B) clustered distinctly (Fig. 1). Similarly, members (pvs. *graminis*, *poae*, *phlei*, and *phleipratensis*) of *X. graminis* sp. nov. clustered distinctly (bootstrap value of 99%) from the other pathovars (Fig. 1).

**Fig. 2.**
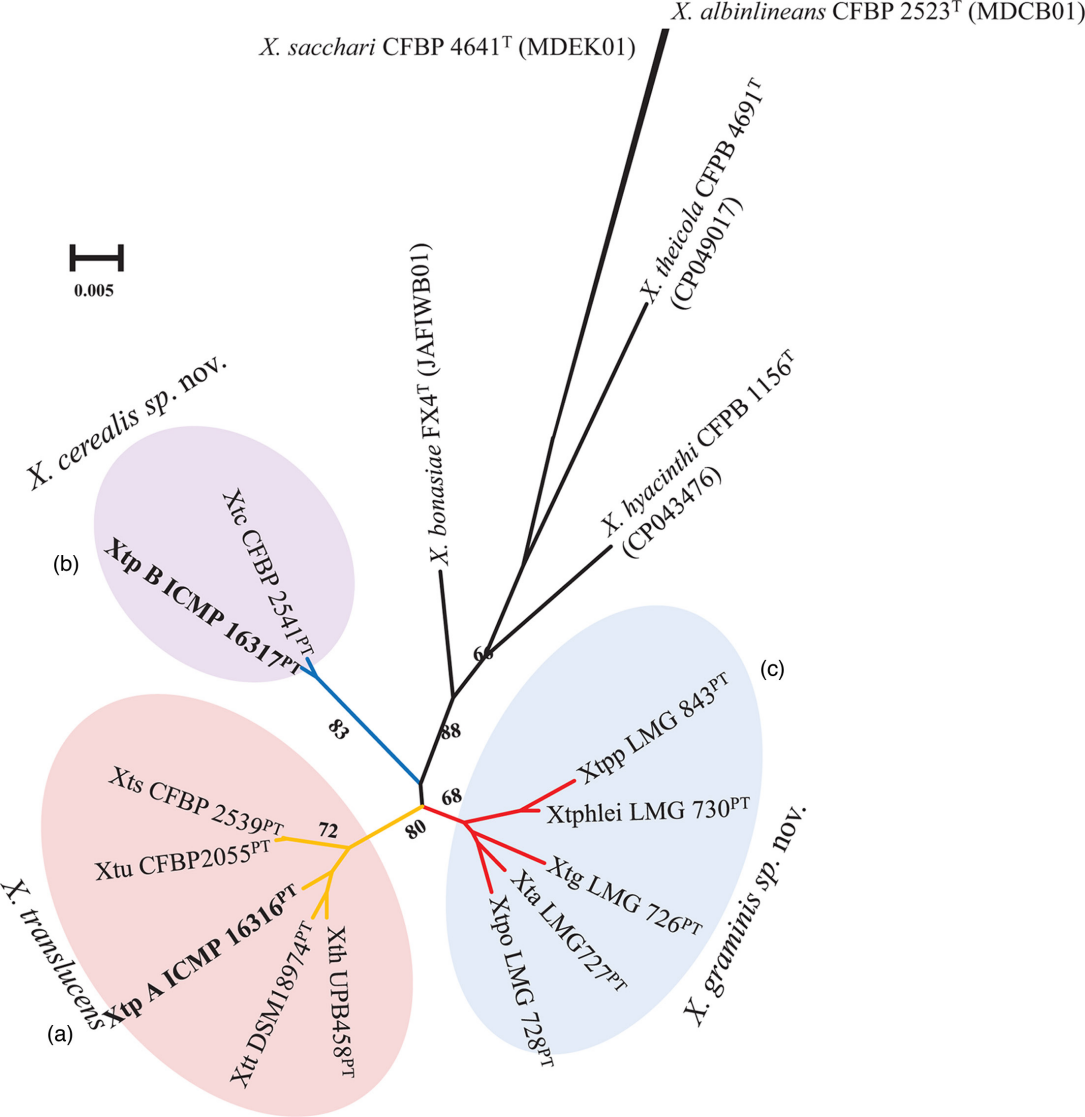
Maximum likelihood tree based on 81 up-to-date bacteria core genes (ubcg2; [46]) confirmed taxonomic delineation of three species. (**a**) *Xanthomonas translucens* (Xtt, pv. *translucens* DSM 18974^PT^; Xth, pv. *hordei* UPB458 ; Xtp, pv. *pistaciae* A CFBP 8304 (= ICMP 16316); Xtu, pv. *undulosa* CFBP 2055^PT^; Xts, pv. *secalis* CFBP 2539). (**b**) *X*. *graminis* sp. nov. (Xtg, pv. *graminis* LMG 726^PT^; Xtpo, pv. *poae* LMG 728^PT^ ; Xta, pv. *arrhenatheri* LMG 727^PT^; Xphlei, pv. *phlei* LMG 730^PT^; Xtpp, pv. *phleipratensis* LMG 843^PT^); and (**c**) *X. cerealis* sp. nov. (Xtc, pv. *cerealis* CFPB 2541^PT^; Xtp B, pv. *pistaciae* strain B ICMP 16317^PT^.

To further validate the DNA–DNA taxonomic relatedness of the pathovars, the 81 ubcg2 pipeline [46] was used to confirm the phylogenomic clustering. The maximum-likelihood ubcg2 tree validated the new proposed taxonomic positions of members of the *X. translucens* complex: ‘true’ *Xanthomonas translucens* (cluster A) and the newly proposed species *X. cerealis* sp. nov. (cluster B) and *X. graminis* sp. nov. (cluster C) (Fig. 2). The three-species concept is in agreement with Goettelmann *et al*. [36] that the *X. translucens* complex consists of three genetically distinct clades based on ANI data alone. Using a suite of genome-based parameters including phylogenomics and dDDH, Tambong *et al*. [37] concluded that the three clades are not only genetically distinct but taxonomically unique. Here, we describe two new species, *X. cerealis* sp. nov. and *X. graminis* sp. nov.

### Orthologous gene cluster analysis and pathogenicity of pv. *pistaciae* strains A and B

**Fig. 3.**
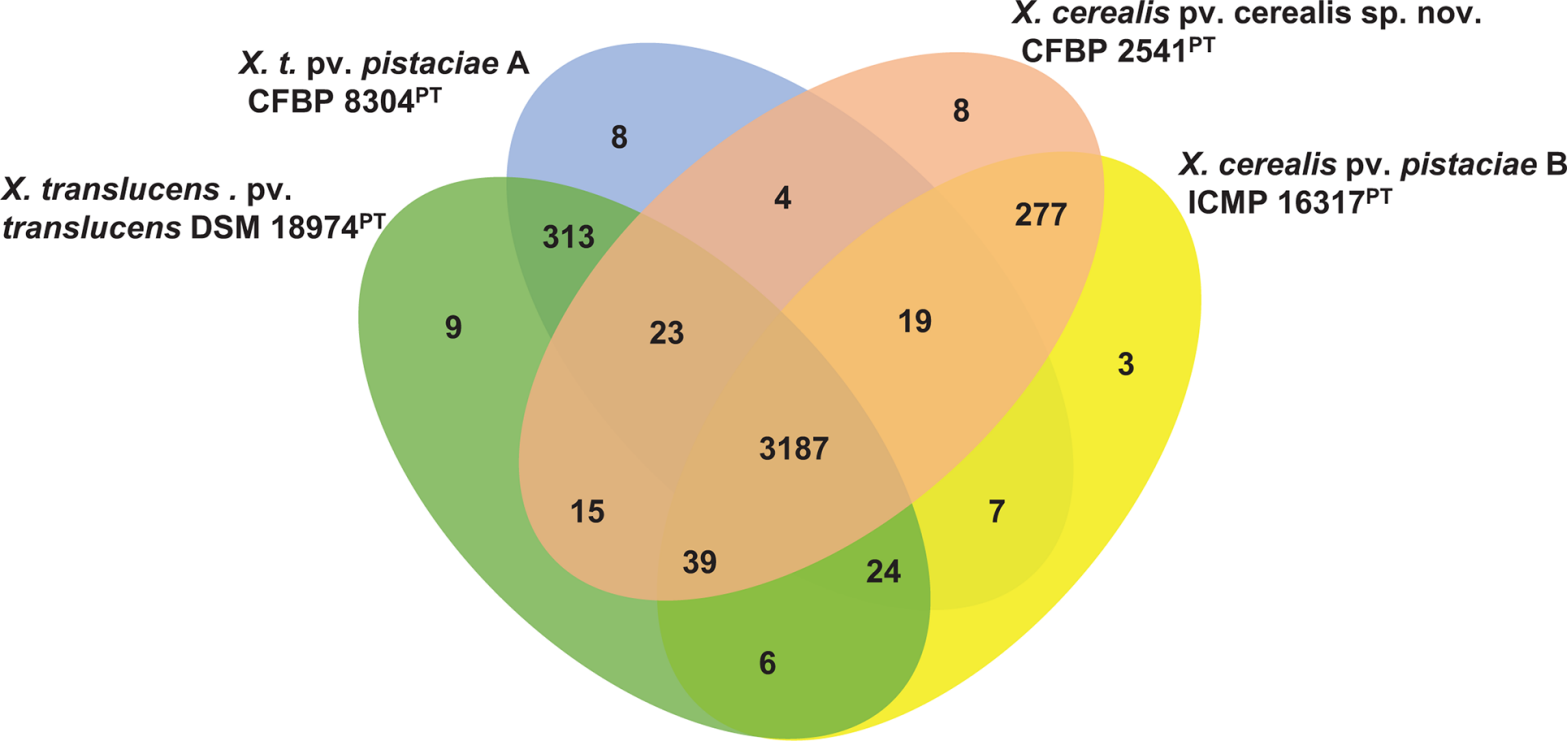
Venn diagram showing shared and unique orthologous protein family clusters of the two strains of pv. *pistaciae* A and B relative to *Xanthomonas translucens* pv. *translucens* and *X. cerealis* pv. *cerealis* sp. nov. Analysis was performed using OrthoVenn3 [49].

Additional insight was gained by analysing the gene content of pv. *pistaciae* strains A and B. Orthologous gene cluster analysis was performed relative to closest species, *X. t*. pv. *translucens* and *X. cerealis* pv. *cerealis* sp. nov. A total of 15 439 proteins were assigned to 3942 orthologous gene clusters with 3156 single-copy clusters and 797 singletons. The four strains shared 3187 protein family clusters (Fig. 3). *X. t*. pv. *translucens* shared the highest number of protein family clusters with pv. *pistaciae* strain A (313) while *X. cerealis* pv. *cerealis* shared the highest protein family cluster counts of 277 with pv. *pistaciae* strain B (Fig. 3), suggesting closer relatives, respectively. A number of unique protein family cluster counts ranging from three (pv. *pistaciae* strain B) to nine (pv. *translucens*) were identified (Fig. 3). blastp analysis of one of the eight unique protein families (cluster 49) showed 88.1% similarity to a putative transposase found in *Xanthomonas fragariae*. Of the three unique protein families (Fig. 3) identified in the genome of pv. *pistaciae* strain B, cluster 30 exhibited 97.5 and 95.9% similarity with a DUF3304 domain-containing protein family of *Xanthomonas hortorum* and *Xanthomonas oryzae*, respectively. This could suggest potential horizontal gene transfers between different *Xanthomonas* species.

**Fig. 4.**
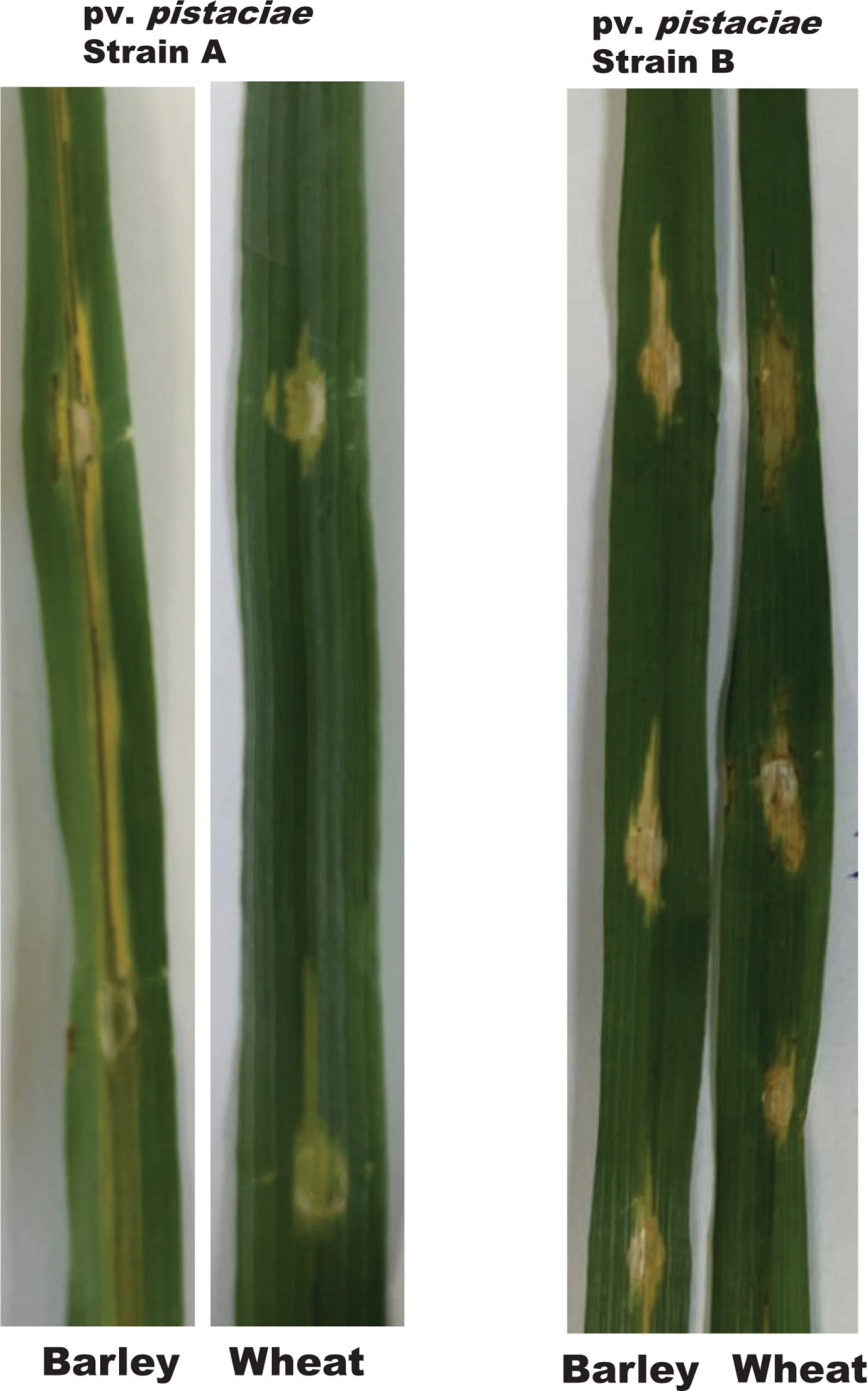
Pathogenicity tests of *Xanthomonas translucens* pv. *pistaciae* strain A (Xtp-A) and *X. cerealis* pv. *pistaciae* strain B (Xcp-B) on barley and wheat confirmed their new species taxonomic and genetic affiliations. Xtp-A caused typical symptoms on barley and not wheat similar to pv. *translucens* while Xcp-B exhibited mild symptoms on both plant types similar to pv. *cerealis* as reported by Bragard *et al*. [16].

In addition, the barley-wheat pathogenicity patterns of pv. *pistaciae* strains A and B match those of their respective assigned closest species. Pv. *pistaciae* strain A which is taxonomically similar to pv. *translucens* (a barley-specific pathovar) induced typical pathogenic symptoms on barley but not on wheat in growth chamber inoculations after 7 days (Fig. 4a). The pathogenicity data suggests that strain A is a putative member of pv. *translucens* and the current pathovar assignment could have been avoided if a host range study that included barley was conducted. In contrast, pv. *pistaciae* strain B induced mild responses on barley cv. CH2909.162.95 and wheat cv. Hoffman (Fig. 4b). Pv. *cerealis* is reported to cause disease on wheat, barley, oat, triticale, and rye [12] and pv. *pistaciae* strain B exhibited mild but observable pathogenic reactions on wheat and barley. This suggests that pv. *pistaciae* strain B is similar to pv. *cerealis*. A known limitation of plant pathogenicity studies is that it is highly influenced by the degree or level of tolerance/resistance of the cultivar or variety. This might have led to the mild pathogenic reactions of barley cv. CH2909.162.95 and wheat cv. Hoffman in response to pv. *pistaciae* strain B infection.

### Phenotypic fingerprinting and fatty acid analysis

Based on Biolog GENIII MicroPlate and API ZYM assays, the proposed novel species could be differentiated from *X. t*. pv. *translucens* by their ability to utilize different carbon sources, enzymatic activity and growth under different pH levels and antimicrobial compounds (chemical sensitivity) (Table 2). The proposed *X. graminis* sp. nov. did not exhibit positive lipase and α-galactosidase activity compared to the other species (Table 2). All strains did not grow at 8% salt concentration, but grew at pH 6; and only *X. translucens* pv. *translucens* grew under a more acidic condition of pH 5. In addition, all the strains with the exception of pv. *translucens* were sensitive to guanidine HCl. Based on Biolog GEN III, only Xtg could not assimilate lactose and malic acid but all the other strains did. Also, Xtc LMG 679^PT^ was the only strain that was unable to utilize d-arabitol (Table 2).

**Table 2.** Host range, carbon utilization, chemical sensitivity and enzymatic activity that differentiate *Xanthomonas graminis pv. graminis sp.* nov. LMG 726 (Xtg), *X. cerealis* pv. *cerealis* sp. nov. LMG 679 (Xtc) and *X. translucens* pv. *translucens* LMG 876 (Xtt) +, Positive reaction; −, negative reaction; w, weakly positive reaction in replicate MicroPlates.

Chemical/reaction	Xtg	Xtc	Xtt
**Host range**	Various grasses	Various cereals	Barley
**API ZYM**			
Enzymatic activity:			
Lipase	+	w	+
β-Galactosidase	−	+	+
α-Galactosidase	−	+	+
**Biolog GEN III:**			
pH 6	+	+	+
pH 5	−	−	+
Chemical sensitivity:			
d-serine	w	−	+
Troleandomycin	w	+	+
Minocycline	−	−	−
Guanidine HCl	−	−	+
Rifamycin SV	−	+	+
Tetrazolium violet	w	w	+
Sodium butyrate	−	+	+
Carbon assimilation:			
Lactose	−	+	+
d-Arabitol	−	+	−
l-Galactonic acid lactone	−	+	+
Propionic acid	+	+	+
Tween 40	w	+	+
d-Malic acid	−	+	+
d-Fucose	−	w	−

Table S1 shows the whole cell fatty acids content of the representative strains which were consistent with those of *sensu stricto* xanthomonads [55,56]. The major (>2 %) cellular fatty acids peaks (Table S1) were C_15 : 0_ iso, C_16 : 1_ ω7*c*/C_16 : 1_ ω6*c* (summed feature 3), C_16 : 0_ 10-methyl or C_17 : 1_ iso ω9*c* (summed feature 9), C_17 : 0_ iso, C_15 : 0_ anteiso, C_16 : 1_ ω9*c*, C_16 : 0_ iso, C_16 : 0_, C_11 : 0_ iso, and C_13 : 0_ iso 3OH, of which the last three are useful for differentiation of xanthomonads from other bacteria [59,60].

## Conclusion

We used dDDH, ANIb and phylogenomics based on ubcg2 and TyGS pipelines to separate the pathovars of the *X. translucens* complex into three distinct species: *X. translucens* (pvs. *translucens* (=*hordei*), *undulosa* (=*secalis*)*,* and *pistaciae* strain A), *X. cerealis* sp. nov. (pvs. *cerealis* and *pistaciae* strain B); and *X. graminis* sp. nov. (pvs. *graminis*, *arrhenatheri*, *poae*, and *phlei (=phleipratensis*)). The assignment of pv. *pistaciae* strains A and B into two different species was strongly supported by their respective barley-wheat pathogenicity patterns as well as orthologous gene analyses. Based on the data presented in this study, seven pathovars (pvs. *translucens*, *undulosa*, *cerealis*, *graminis*, *arrhenatheri*, *poae*, and *phlei*) remain relevant but separated into three species. This new taxonomic assignment might provide a different framework for determining the quarantine status of the old members of the *X. translucens* complex by regulatory organisations such as EPPO. This could facilitate rapid international exchange of barley and wheat germplasm. Based on analyses of the phenotypic, biochemical, phylogenomic, dDDH and ANIb data, we propose two new species, *Xanthomonas cerealis* sp. nov. and *Xanthomonas graminis* sp. nov.

## Description of *Xanthomonas cerealis* sp. nov.

*Xanthomonas cerealis* (ce.re.a’lis. L. fem. adj. *cerealis*, related to cereal crops).

The characteristics of this new species are as described for the genus by [54] extended with data generated from our study. Cells are obligately aerobic, Gram-reaction-negative, straight rods. The major (>2%) cellular fatty acids peaks are C_15 : 0_ iso, C_16 : 1_ ω7*c*/C_16 : 1_ ω6*c* (summed feature 3), C_16 : 0_ 10-methyl or C_17 : 1_ iso ω9*c* (summed feature 9), C_17 : 0_ iso, C_15 : 0_ anteiso, C_16 : 1_ ω9*c*, C_16 : 0_ iso, C_16 : 0_, C_11 : 0_ iso, C_13 : 0_ iso 3OH. It grew at NaCl concentrations of 1 and 4%, but not at 8%. Growth was observed at pH 6 but not at acidic pH of 5. Based on Biolog GENIII MicroPlate assays, strain LMG 679^T^ utilizes 45 carbon sources: cellobiose, sucrose, dextrin, trehalose, lactose, d-salicin, *N*-acetyl-d-glucosamine,α-d-glucose, d-mannose, d-fructose, d-galactose, 3-methyl glucose, d-fucose, l-fucose, l-rhamnose, d-arabitol, glycerol, d-glucose-6-PO_4_, d-fructose-6-PO_4_, gelatin, glycyl-l-proline, l-alanine, l-arginine, l-aspartic acid, l-glutamic acid, l-serine, pectin, d-galacturonic acid, l-galactonic acid, lactone, glucuronamide, d-saccharic acid, methyl pyruvate, α-keto-glutaric acid, d-malic acid, l-malic acid, bromo-succinic acid, Tween 40, α-hydroxy-butyric acid, l-lactic acid, citric acid, β-hydroxy-d,l-butyric acid, propionic acid, acetic acid,α-keto-butyric acid, and formic acid. The strain does not utilize 23 carbon sources: turanose, stachyose, gentiobiose, maltose, raffinose, methyl β-d-glucoside, *N*-acetyl neuraminic acid, *N*-acetyl-d-galactosamine, inosine, d-sorbitol, d-aspartic acid, d-serine, d-mannitol, *myo*-inositol, l-pyroglutamic acid, mucic acid, quinic acid, d-gluconic acid, d-glucuronic acid, *p*-hydroxy-phenylacetic acid, d-lactic acid, methyl ester, γ-amino-butryric acid, and acetoacetic acid. This bacterium is resistant to troleandomycin, rifamycin SV, vancomycin, lincomycin, 1% sodium lactate, fusidic acid, potassium tellurite, tetrazolium violet, and tetrazolium blue but sensitive to nalidixic acid, minocycline, d-serine, guanidine HCl, niaproof 4 and lithium chloride. Using API ZYM assays, this bacterium is positive for esterase, esterase, alkaline phosphatase, lipase, leucine arylamidase, cystine arylamidase, valine arylamidase, trypsin, α-chymotrypsin, acid phosphatase, naphthol-AS-Bl-phosphaohydrolase, α-galactosidase, β-galactosidase,β-glucosidase, *N*-acetyl-β-glucosaminidase but negative for lipase, β-glucuronidase, α-glucosidase, α-fucosidase, and α-mannosidase.

The type strain is LMG 679^T^ (=NCPPB 1944^T^), isolated from *Bromus inermis* in the USA. The DNA G+C content of LMG 679^T^ is 67.5 mol%. The GenBank whole-genome sequence accession number is CP074364.

Based on plant host pathogenicity, two pathovars are identified:

***Xanthomonas cerealis*** **pv.** ***cerealis*** **(Hagborg 1942) Vauterin** ***et al*****. 1995.**

= *Xanthomonas translucens* pv. *cerealis* (Hagborg 1942) Vauterin *et al*. 1995.

The GenBank whole-genome sequence accession number is CP074364.

=*Xanthomonas translucens* pv. *pistaciae* strain B (Hagborg 1942; Vauterin *et al*. 1995) Giblot-Ducray *et al*. 2009. The GenBank whole-genome sequence accession number is CP083804.

## Description of *Xanthomonas graminis* sp. nov.

*Xanthomonas graminis* (gra′mi.nis. L. gen. n. *graminis*, of grass).

The characteristics of this new species are as previously described for the genus by Vauterin *et al*. [54] extended with data generated from our study. Cells are obligate, aerobic, Gram-reaction-negative, straight rods. The major (>2%) cellular fatty acids peaks are C_15 : 0_ iso, C_16 : 1_ ω7*c*/C_16 : 1_ ω6*c* (summed feature 3), C_16 : 0_ 10-methyl or C_17 : 1_ iso ω9*c* (summed feature 9), C_17 : 0_ iso, C_15 : 0_ anteiso, C_16 : 1_ ω9*c*, C_16 : 0_ iso, C_16 : 0_, C_11 : 0_ iso, and C_13 : 0_ iso 3OH. It grows at NaCl concentrations of 1 and 4%, but not at 8%. Growth is observed at pH 6 but not at acidic pH of 5. Based on Biolog GEN III MicroPlate assays, strain LMG 726^T^ utilized 36 carbon sources: cellobiose, sucrose, maltose, trehalose, d-salicin, *N*-acetyl-d-glucosamine, *N*-acetyl-β-d-mannosamine, α-d-glucose, d-mannose, d-fructose, d-galactose, 3-methyl glucose, glycerol, d-glucose-6-PO_4_, d-fructose-6-PO_4_, gelatin, l-alanine, l-aspartic acid, l-glutamic acid, l-serine, pectin, glucuronamide, methyl pyruvate, l-lactic acid, citric acid, α-keto-glutaric acid, l-malic acid, bromo-succinic acid, Tween 40, α-hydroxy-butyric acid, propionic acid, acetic acid β-hydroxy-d,l-butyric acid, α-keto-butyric acid, and acetoacetic acid. The strain does not utilize 37 carbon sources: gentiobiose, turanose, raffinose, lactose stachyose, melibiose, *N*-acetyl-d-galactosamine, *N*-acetyl neuraminic acid,β-methyl-d-glucoside, d-fucose, inosine, d-sorbitol, d-mannitol, d-arabitol, *myo*-inositol, d-aspartic acid, d-serine, l-arginine, l-histidine, l-pyroglutamic acid, lactone, d-gluconic acid, d-galacturonic acid, l-galactonic acid, d-glucuronic acid, d-saccharic acid, *p*-hydroxy-phenylacetic acid, mucic acid, quinic acid, d-lactic acid, methyl ester, d-malic acid, γ-amino-butryric acid, l-fucose, l-rhamnose, inosine and acetic acid. It is resistant to 1% sodium lactate, fusidic acid, d-serine, tetrazolium violet, tetrazolium blue, troleandomycin, rifamycin SV, lincomycin, vancomycin, lithium chloride, and aztreonam and but sensitive to 1% sodium lactate, fusidic acid, sodium bromate, sodium butyrate, minocycline, nalidixic acid, guanidine HCl, niaproof 4 and potassium tellurite. Using API ZYM assay, these bacteria are positive for esterase, esterase lipase, lipase, leucine arylamidase, alkaline phosphatase, cystine arylamidase, valine arylamidase, trypsin, α-chymotrypsin, β-glucosidase, *N*-acetyl-β-glucosaminidase, acid phosphatase, and naphthol-AS-Bl-phosphaohydrolase but negative for α-galactosidase, β-galactosidase, α-fucosidase,α-glucosidase, β-glucuronidase, and α-mannosidase.

The type strain is LMG 726^T^ (=NCPPB 2700^T^) isolated from *Dactylis glomerata,* near Changins, Switzerland. The genomic DNA G+C content of LMG 726^T^ is 68.0 mol%. The GenBank whole-genome sequence accession number is CP076254.

Based on plant pathogenic differences, four pathovars can be distinguished:

***Xanthomonas graminis* pv. *arrhenatheri* (Egli & Schmidt 1982) Vauterin, Hoste, Kersters and Swings 1995**

*= Xanthomonas translucens* pv. *arrhenatheri* (Egli & Schmidt 1982) Vauterin *et al*. 1995.

Pathotype strain: LMG 727=NCPPB 3229

The GenBank whole-genome sequence accession number is CP086333.

***Xanthomonas graminis*** **pv.** ***graminis*** **(Egli** ***et al*****. 1975) Vauterin, Hoste, Kersters and Swings 1995**

= *Xanthomonas translucens* pv. *graminis* (Egli *et al*. 1975) Vauterin *et al*. 1995

Other name : *Xanthomonas campestris* pv. *graminis* (Egli *et al*. 1975)

Pathotype strain: LMG 726=NCPPB 2700.

The GenBank whole-genome sequence accession number is CP076254.

***Xanthomonas graminis*** **pv.** ***phlei*** **(Wallin & Reddy) Vauterin, Hoste, Kersters and Swings 1995**

= *Xanthomonas translucens* pv. *phlei* (Wallin and Reddy) Vauterin *et al*. 1995

Other name : *Xanthomonas campestris* pv. phlei (Wallin and Reddy)

Pathotype strain: LMG 730=NCPPB 3231. The GenBank whole-genome sequence accession number is CP076251.

=*Xanthomonas translucens* pv. *phleipratensis* (Wallin and Reddy) Vauterin *et al*. 1995

Other name: *Xanthomonas campestris* pv. *phleipratensis* (Wallin and Reddy)

Pathotype strain: LMG 843=NCPPB 1837. The GenBank whole-genome sequence accession number is CP086332.

***Xanthomonas graminis*** **pv. *poae* (Egli & Schmidt 1982) Vauterin, Hoste, Kersters and Swings 1995**

*=Xanthomonas translucens* pv. *poae* (Egli & Schmidt 1982) Vauterin *et al*. 1995.

Other scientific name: *Xanthomonas campestris* pv. *poae* (Egli & Schmidt 1982).

Pathotype strain: LMG 728=NCPPB 3230. The GenBank whole-genome sequence accession number is CP076250.

## Supplementary material

10.1099/ijsem.0.006523 Uncited Supplementary Material 1.

## Abbreviations

ANIb: blast-based average nucleotide identity
BDD: bacterial dieback disease
BLS: bacterial leaf streak
dDDH: digital DNA–DNA hybridization
EPPO: European and Mediterranean Plant Protection Organization
LB: Luria–Bertani agar medium
ML: maximum-likelihood
MLSA: multilocus sequence analysis
TyGS: Type Strain Genome Server
ubcg2: up-to-date bacterial core gene set version 2
wDDH: wet-laboratory DNA-DNA hybridization
Xtc: *Xanthomonas translucens* pv. *cerealis*
Xtg: *Xanthomonas translucens* pv. *graminis*
Xtt: *Xanthomonas translucens* pv. *translucens*
