# Supplementary material for: Taxonogenomic analysis of the Xanthomonas translucens complex leads to the descriptions of Xanthomonas cerealis sp. nov. and Xanthomonas graminis sp. nov
Source: Int J Syst Evol Microbiol. 2024 Sep 19;74(9):006523. doi: 10.1099/ijsem.0.006523 (PMC12453568; doi:10.1099/ijsem.0.006523)
Supplement: Uncited Supplementary Material 1. [file ijsem-74-06523-s001.pdf]

Unclassified / Non classifié

Table S1. Cellular fatty acid composition (%) of *Xanthomonas graminis sp.* nov. (Xtg), *X. cerealis sp.* nov. (Xtc) and *X. translucens* pv. *translucens* (Xtt)[a]

**Carbon chain/radicals**	**Xtg**	**Xtc**	**Xtt**
$C_{10:0}$	0.76	0.48	0.49
$C_{11:0}$ iso	4.74	5.36	5.40
$C_{11:0}$ iso 3OH	3.32	3.49	3.20
$C_{12:0}$ 3OH	1.82	1.71	1.76
$C_{13:0}$ iso 3OH	2.38	2.15	2.35
$C_{14:0}$ iso	0.63	0.67	0.68
$C_{14:0}$	0.63	1.27	1.31
$C_{15:1}$ iso F	0.91	0.71	0.60
$C_{15:0}$ iso	20.68	29.30	26.38
$C_{15:0}$ anteiso	6.64	4.95	6.66
$C_{15:1}$ ω6c	nd	nd	nd
$C_{16:0}$ iso	2.28	2.34	3.43
$C_{16:1}$ ω9c	3.40	1.94	1.90
$C_{16:0}$	4.00	5.14	5.99
$C_{17:0}$ iso	9.60	5.52	6.31
$C_{17:0}$ anteiso	0.90	0.34	0.59
$C_{17:1}$ ω8c	0.76	0.58	0.63
$C_{18:1}$ ω9c	2.00	0.38	0.48
$C_{17:0}$ iso 3OH	0.53	nd	nd
$C_{16:1}$ ω7c / $C_{16:1}$ ω6c[b]	19.05	21.72	20.92
$C_{17:1}$ iso I / anteiso B[c]	0.82	nd	nd
$C_{18:1}$ ω7c[d]	0.95	nd	0.18
$C_{16:0}$ 10-methyl or $C_{17:1}$ iso ω9c[e]	11.03	10.01	8.46

[a]Extraction of fatty acids and analysis were performed by Keystone Laboratory (Alberta, Canada) using the standard MIDI System protocol. Fatty acids with amounts < 0.50% across bacterial strains are not reported. nd, not detected. [b] summed feature 3; [c] summed feature 4; [d] summed feature 8; and [e] summed feature 9.

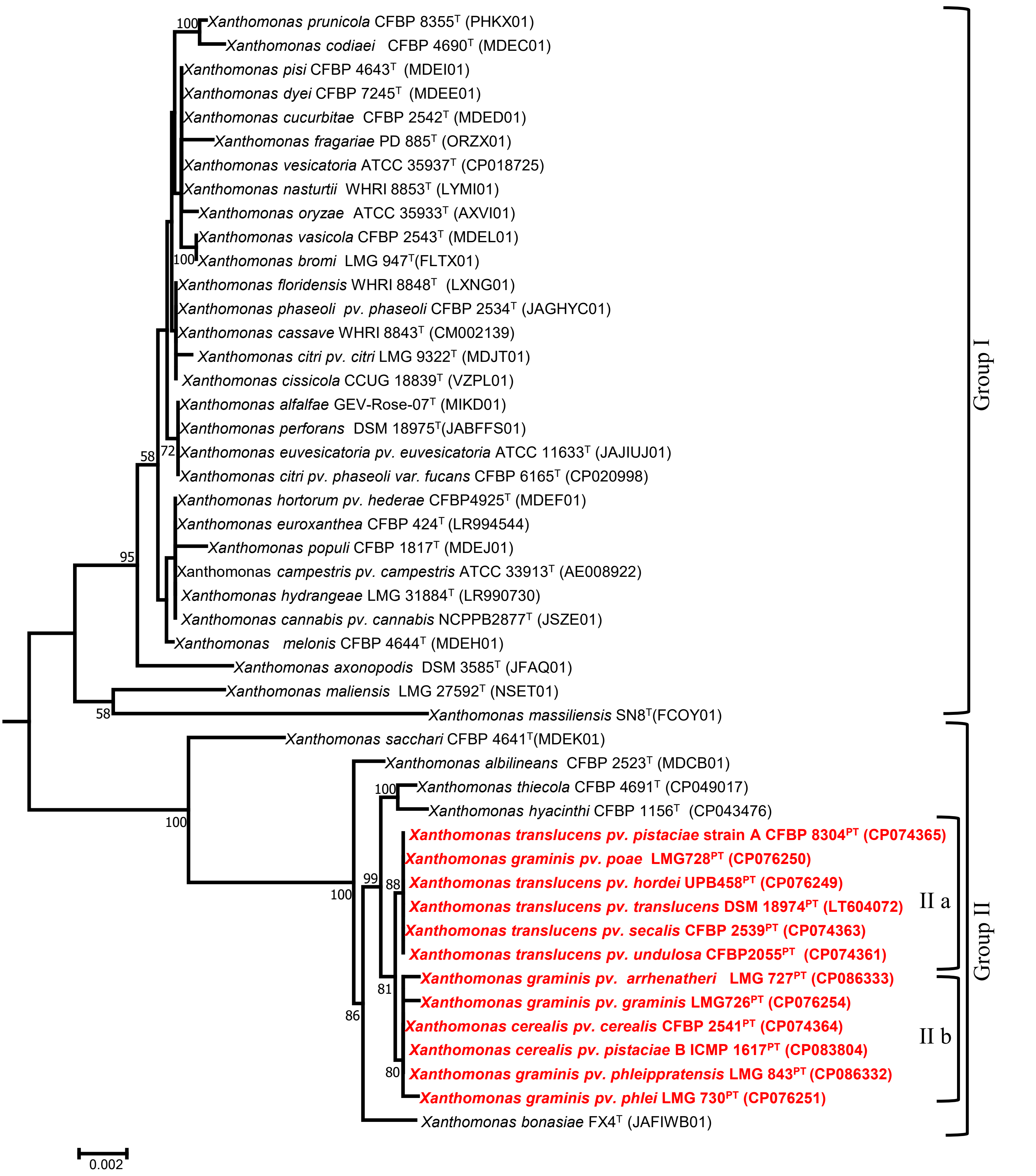

Figure S1. 16S rRNA-based phylogenetic tree showing the 11 pathovars (red) of the *X. translucens* complex clustering relative to 46 species of the genus *Xanthomonas*. Bootstrap values > 50% are shown at the nodes.
